# Phytol derived from chlorophyll hydrolysis in plants is metabolized *via* phytenal

**DOI:** 10.1016/j.jbc.2021.100530

**Published:** 2021-03-11

**Authors:** Philipp Gutbrod, Wentao Yang, Goran Vuk Grujicic, Helga Peisker, Katharina Gutbrod, Lin Fang Du, Peter Dörmann

**Affiliations:** 1 Institute of Molecular Physiology and Biotechnology of Plants (IMBIO), University of Bonn, Bonn, Germany; 2 Key Laboratory of Bio-Resources and Eco-Environment of the Ministry of Education, College of Life Sciences, Sichuan University, Chengdu, China

**Keywords:** *Arabidopsis*, chlorophyll, plant biochemistry, lipid oxidation, stress, vitamin E, aldehyde, phytol, FAPE, fatty acid phytyl ester, MRM, multiple reaction monitoring, MS, Murashige and Skoog, phytyl-PP, phytyl-diphosphate, Q-TOF, quadrupole-TOF, SPE, solid-phase extraction

## Abstract

Phytol is the isoprenoid alcohol bound in ester linkage to chlorophyll, the most abundant photosynthetic pigment in plants. During leaf senescence, large amounts of phytol are released by chlorophyll degradation. However, the pathway of phytol catabolism in plants is unknown. We hypothesized that phytol degradation in plants might involve its oxidation into the long-chain aldehyde phytenal. Using GC-MS for aldehyde quantification after derivatization with methylhydroxylamine, phytenal was identified in leaves, whereas other long-chain aldehydes (phytanal and pristanal) were barely detectable. We found that phytenal accumulates during chlorotic stresses, for example, salt stress, dark-induced senescence, and nitrogen deprivation. The increase in the phytenal content is mediated at least in part independently of enzyme activities, and it is independent of light. Characterization of phytenal accumulation in the *pao1* mutant affected in chlorophyll degradation revealed that phytenal is an authentic phytol metabolite derived from chlorophyll breakdown. The increase in phytenal was even stronger in mutants affected in the production of other phytol metabolites including *vte5-2* (tocopherol deficient) and *pes1 pes2* (fatty acid phytyl ester deficient). Therefore, phytenal accumulation is controlled by competing, alternative pathways of phosphorylation (leading to tocopherol production) or esterification (fatty acid phytyl ester production). As a consequence, the content of phytenal is maintained at low levels, presumably to minimize its toxic effects caused by its highly reactive aldehyde group that can form covalent bonds with and inactivate the amino groups of proteins.

Chlorophyll is the most important photosynthetic pigment in plants, and it is crucial for light harvesting and channeling of photons to the reaction centers of photosystems I and II. Chlorophyll is subject to constant turnover, and chlorophyll degradation is stimulated during stress or senescence (1). The degradation of chlorophyll by enzymes of chlorophyll catabolism is important as some chlorophyll catabolites can produce radicals after illumination, which are highly toxic to the cell (1).

The chlorophyll molecule is composed of the chlorophyllide head group derived from the porphyrin biosynthetic pathway and the phytyl side chain from the isoprenoid pathway. The phytyl chain is linked in an ester bond to the carboxylate group of the chlorophyllide, and it can be hydrolyzed by pheophytin pheophorbide hydrolase or chlorophyll dephytylase 1 during chlorophyll breakdown (2, 3). Phytol serves as a precursor for the synthesis of different chloroplast lipids. It can be converted into fatty acid phytyl esters (FAPEs), which accumulate in the plastoglobules of chloroplasts during stress (4, 5). Two acyltransferases, PES1 and PES2, are involved in transferring activated fatty acids onto free phytol, thereby producing FAPEs in *Arabidopsis* (6). Alternatively, free phytol can be phosphorylated by phytol kinase (VTE5) yielding phytyl phosphate, which can be further phosphorylated to phytyl diphosphate (phytyl-PP) by VTE6 (7, 8). Phytyl-PP is the substrate for the synthesis of tocopherol (vitamin E) and phylloquinone (vitamin K1) (9, 10). Because phytyl-PP derived from phytol phosphorylation is the main source for tocopherol synthesis, the *vte5* and *vte6* mutants of *Arabidopsis* are tocopherol deficient.

The pathway of phytol degradation in plants is largely unknown. In humans, phytol taken up from the diet is converted into phytenal and phytanoyl-CoA, which is degraded *via* α- and β-oxidation including the synthesis of the intermediate pristanal, another C19 isoprenoid aldehyde (Fig. 1) (11). Phytanoyl-CoA has been detected in *Arabidopsis*, suggesting that a pathway similar to the human pathway might also exist in plants (12, 13). In addition, phytenal was tentatively identified in senescent oat leaves (14). The conversion of phytol into phytenal was suggested to be mediated *via* photooxidation (14). The analysis of the phytol degradation pathway is hampered by the fact that phytol catabolites are extremely low abundant and highly unstable. The analysis of long-chain aldehydes such as phytenal, phytanal, and pristanal is challenging because they are prone to oxidation during extraction (14).

**Figure 1:**
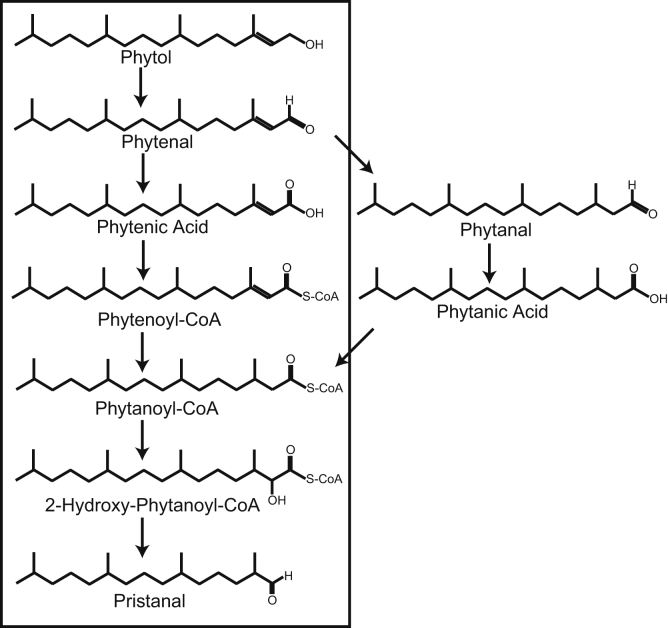
**Hypothetical pathway of phytol degradation in plants.** In mammals, phytol derived from chlorophyll taken up through the diet is degraded *via* phytenal and phytenic acid. Phytenic acid is converted into phytenoyl-CoA, phytanoyl-CoA, 2-hydroxy-phytanoyl-CoA, and pristanal. Although the mammalian pathway which presumably also exists in plants is framed with a box, clear evidence for the involvement of phytanal or free phytanic acid in phytol degradation is lacking.

To study phytol metabolism in plants, highly sensitive methods for quantification of phytenal and other long chain aldehydes based on GC-MS and LC-MS were used. Analysis of leaves form *Arabidopsis* plants revealed that phytenal can be detected and represents the most abundant long-chain aldehyde. Other long-chain aldehydes, including phytanal and pristanal, are barely detectable in leaves. The measurements of phytenal in WT and mutant plants of *Arabidopsis* grown under control and abiotic stress conditions indicated that phytenal represents an authentic intermediate of phytol catabolism.

## Results

### Quantification of phytenal in plants by GC-MS

**Figure 2:**
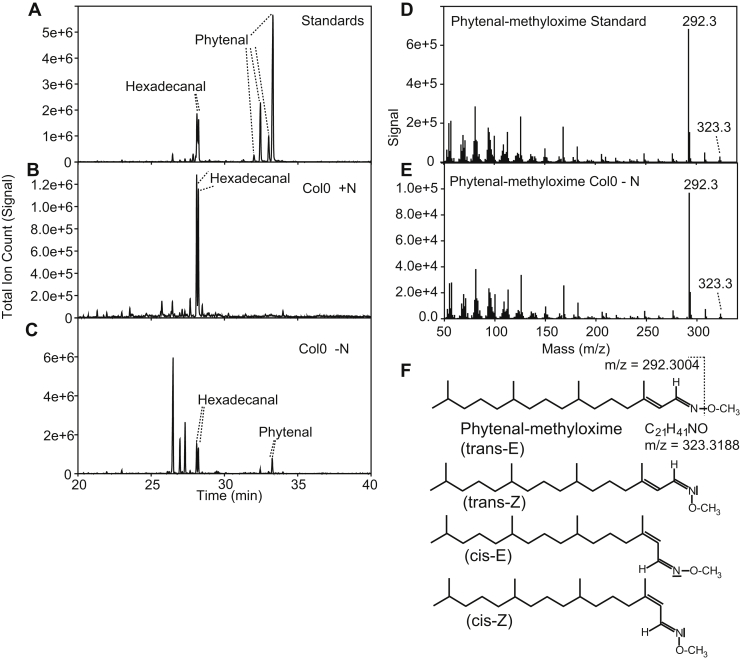
**GC-MS separation of aldehyde-methyloximes from whole *Arabidopsis* leaves.** Aldehyde standards or aldehydes extracted from *Arabidopsis* leaves were derivatized with methylhydroxylamine and separated by GC-MS. The panels show GC-MS total ion count (TIC) chromatograms or mass spectra of individual peaks. *A*, the chromatogram of the standards hexadecanal–methyloxime and phytenal–methyloxime. *B*, the chromatogram of derivatized aldehydes from green *Arabidopsis* WT leaves. *C*, the chromatogram of derivatized aldehydes from leaves of *Arabidopsis* WT plants after nitrogen deprivation. *D*, mass spectrum of phytenal–methyloxime standard (peak at 33.3 min in panel *A*). *E*, mass spectrum of and phytenal-methyloxime from leaves (peak at 33.3 min in panel *C*). *F*, structure of phytenal-methyloxime indicating the major fragment of m/z 292.3 originating from the loss of the O-CH_3_ group. The authentic plant-derived phytenal carries a *trans*-double bond, while the synthetic phytol used for the synthesis of phytenal standard represents a mixture of *cis*-/*trans*-isomers. Owing to the presence of the free electron pair on the nitrogen, the methyloxime group of all derivatized aldehydes can be in E configuration (preferred) or Z configuration. Note that all isomers of phytenal-methyloxime give rise to the very same mass spectrum.

Aldehyde measurements in plants are complicated by the fact that they are highly reactive and prone to oxidation. Derivatization can protect the functional group of aldehydes, thereby increasing the recovery from plant tissues. We selected methoxylamine as the derivatization reagent because it efficiently converts aldehydes into methyloximes. Methyloximes of long-chain aldehydes are volatile and can be analyzed by GC-MS (15). The analysis of standards of hexadecanal and phytenal revealed two peaks for hexadecanal, and four peaks for phytenal (Fig. 2). The mass spectra of the two hexadecanal peaks were almost identical and showed the molecular ion peak of hexadecanal–methyloxime of 323.3 and an M-31 fragment corresponding to the loss of the methoxy group. It is known that aldehyde–methyloximes form two stereoisomers (E/Z) that differ by the configuration of the methoxy group, which is closer (Z) or further away (E) from the hydrocarbon chain (Fig. 2) (16). Four peaks were identified in the GC-MS chromatogram for phytenal, all showing the same spectrum with the molecular ion peak of 292.3 m/z and an M-31 fragment. The occurrence of four phytenal–methyloxime peaks can be explained by the fact that the phytenal standard represents a mixture of *cis*-/*trans*-isomers, derived from the synthetic phytol used for its synthesis, and each of the two isomers gives rise to E/Z methoxy diastereomers after derivatization (Fig. 2).

Next, a crude lipid extract from green leaves of *Arabidopsis* was derivatized with methoxylamine. However, aldehyde–methyloximes could not be detected *via* GC-MS because of the high background in the chromatograms. We considered the possibility that phytenal, as a possible degradation product of phytol, might accumulate during chlorophyll breakdown and therefore grew plants under nitrogen deprivation to stimulate leaf chlorosis. However, it was still not possible to detect phytenal or other aldehydes after derivatization by GC-MS. In the following, the aldehyde-methyloximes from crude leaf extracts were first purified *via* solid-phase extraction (SPE) on a silica column. Although we could still not detect any aldehyde–methyloximes from green leaves, it was now possible to find phytenal in nitrogen-deprived plants by GC-MS after derivatization and SPE purification (Fig. 2). Phytenal was the only long-chain aldehyde detected in the GC-MS chromatogram of nitrogen-deprived plants. No other straight or branched-chain aldehydes were found.

**Figure 3:**
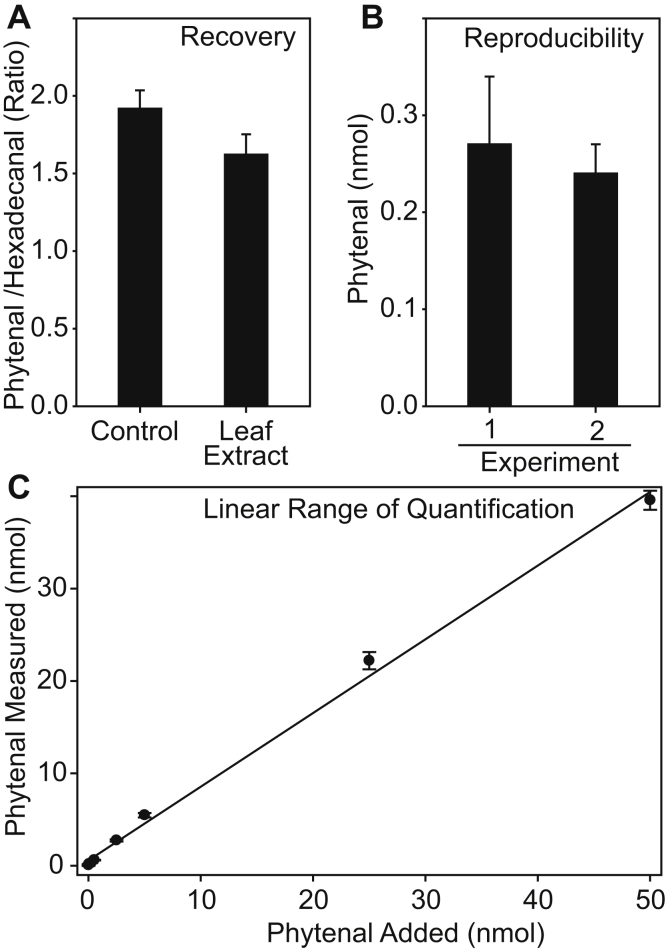
**Recovery, reproducibility, and linear range of quantification of phytenal by GC-MS after derivatization with methyloxime.***A*, the standards phytenal and hexadecanal were mixed in a nmol ratio of 2:1 and after methyloxime treatment, the ratio of the two peak areas quantified by GC-MS (control). The *right bar* shows the ratio of phytenal/hexadecanal peak areas extracted and purified by solid-phase extraction in the presence of lipids from a green leaf. The recovery was calculated as amount of phytenal measured in the presence of leaf lipids *versus* phytenal measured in the absence of leaf lipids and equals 84.6%. (n = 5, mean ± SD). *B*, phytenal was measured in *Arabidopsis* WT leaves with the internal standard hexadecanal. The 2 bars are derived from two different experiments performed on two different days. Reproducibility of the two measurements was 89.0%. (n = 3, mean ± SD). *C*, phytenal in amounts from 0 to 50 nmol was added to a leaf extract and quantified using hexadecanal as the internal standard. The range of linear detection is from ∼0.05 nmol to ∼50 nmol phytenal.

Quantification of phytenal was achieved using hexadecanal as the internal standard. To determine the recovery of phytenal, a known amount of phytenal was spiked into a green leaf extract and after methoxylamine treatment and SPE purification, quantified by GC-MS. The recovery was defined as the amount of phytenal measured in the spiked leaf extract in relation to the amount measured after derivatization of the same amount of phytenal in the absence of the leaf extract and without SPE purification. The recovery was 84.6% (Fig. 3). The reproducibility was determined by quantification of phytenal in two independent experiments on different days. The amount of phytenal measured in the experiments varied by 11%, and this difference was not significant (Fig. 3). Next, we determined the linearity of the GC-MS method by adding different amounts of phytenal to green leaf lipid extracts followed by derivatization, SPE purification, and quantification by GC-MS. The plot of phytenal measured *versus* phytenal added was linear in the range from 0.25 to 50 nmol with an r^2^ of 0.996 (Fig. 3). The detection limit as defined by the signal-to-noise ratio of 3:1 was 0.05 nmol.

### Analysis of long-chain aldehydes from plants by LC-MS

**Figure 4:**
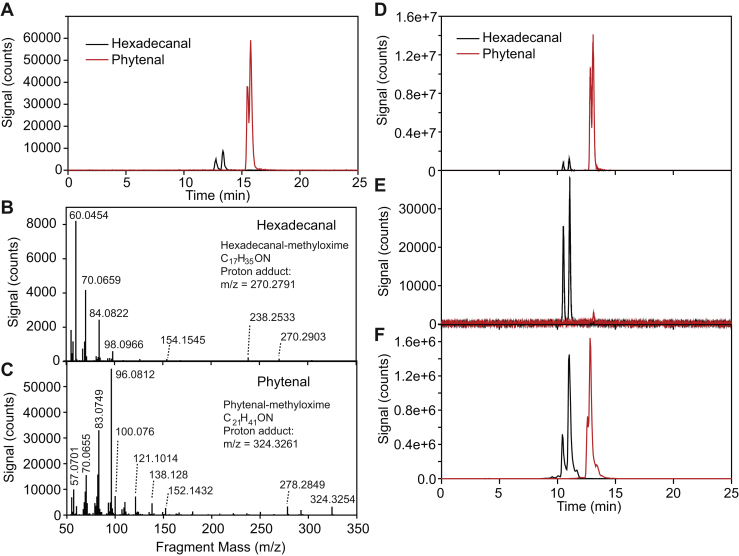
**Identification of phytenal in *Arabidopsis* leaves by LC-MS/MS analysis.** Aldehydes were derivatized with methyloxime and separated by LC-MS/MS. *A*, the extracted ion chromatogram of the separation of the standards hexadecanal–methyloxime (m/z 270.2797, *black*) and phytenal–methyloxime (m/z 324.3266, *red*) by LC-MS/MS on a Q-TOF instrument. *B*, Q-TOF MS/MS spectrum of hexadecanal-methyloxime with fragmentation of m/z 270.2903 eluting at 13.37 min. *C*, Q-TOF MS/MS spectrum of phytenal–methyloxime with fragmentation of m/z 324.3254 eluting at 15.75 min. *D*, the MRM chromatogram of the standards hexadecanal–methyloxime (*black*, transition of 270.3 → 60.0) and phytenal–methyloxime (*red*, transition of 324.3 → 96.0) obtained by LC-MS/MS on a Q-Trap instrument. *E*, the MRM chromatogram of methyloxime-treated aldehydes from green *Arabidopsis* leaves containing hexadecanal standard on a Q-Trap instrument. *F*, the MRM chromatogram of methyloxime-treated aldehydes from leaves of nitrogen-deprived *Arabidopsis* plants measured on a Q-Trap instrument. Note that the presence of E or Z isomers of the aldehyde–methyloximes gives rise to two peaks in the chromatograms with identical mass spectra. MRM, multiple reaction monitoring; Q-TOF, quadrupole-TOF.

To determine whether in addition to phytenal other long-chain aldehydes can be detected in whole-leaf extracts, aldehyde–methyloximes were measured by LC-MS/MS instead of GC-MS. First, a quadrupole-TOF (Q-TOF) instrument with high mass accuracy was used. After derivatization and SPE purification, two peaks each corresponding to the E/Z methyloxime isomers were identified in the Q-TOF chromatogram for the standards of hexadecanal (12.5, 13.0 min) and phytenal (15.1, 15.2 min), each with characteristic mass spectrum showing large fragment peaks at m/z of 60.04 (hexadecanal) and 96.08 (phytenal) (Fig. 4, *A*–*C*).

**Figure 5:**
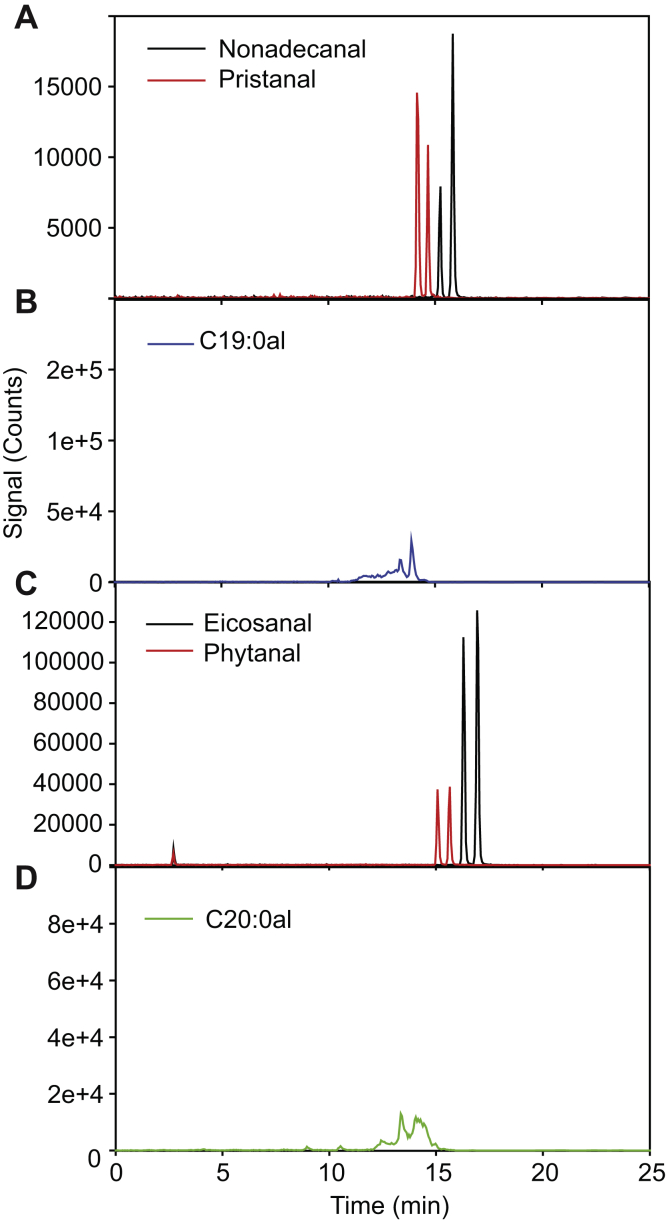
**Analysis of nonadecanal, pristanal, eicosanal, and phytanal by LC-MS/MS.** Aldehydes were derivatized with methyloxime and separated by LC-MS/MS on a Q-Trap instrument. *A*, chromatograms for C19:0 aldehyde–methyloximes (MRM 312 → 60) of nonadecanal (*black*) and pristanal (*red*) standards. *B*, C19:0 aldehydes from *Arabidopsis* WT leaves of plants grown under nitrogen deprivation. Aldehydes were treated with methyloxime and analyzed for the presence of C19 aldehydes (MRM 312 → 60). *C*, chromatograms for C20:0 aldehyde–methyloximes (MRM 326 → 60) of eicosanal (*black*) and phytanal (*red*) standards. *D*, C20 aldehydes from *Arabidopsis* WT leaves of plants grown under nitrogen deprivation. Aldehydes were treated with methyloxime and analyzed for the presence of C20 aldehydes (MRM for 326 → 60). MRM, multiple reaction monitoring.

To increase the sensitivity even further, LC-MS/MS measurements were performed using a highly sensitive triple quadrupole instrument. Multiple reaction monitoring (MRM) transitions for the aldehyde-methyloxime peaks derived from the Q-TOF spectra were 270.28/60.04 for hexadecanal and 324.33/96.08 for phytenal (Fig. 4, *D*–*F*). The LC-MS/MS triple quadrupole method was used to search for other long-chain aldehydes in the whole-leaf extract, that is, even- and odd-chain aldehydes with 10 to 20 carbon atoms, including straight-chain and branched-chain aldehydes derived from acyl or isoprenoid metabolism. The MRM transitions were derived from the mass spectrum of hexadecanal (Fig. 4, Table S1). Figure 5 shows LC-MS/MS chromatograms of pristanal and phytanal standards. The straight-chain isomers of pristanal and phytanal, that is, nonadecanal (19:0al) and eicosanal (20:0al), respectively, could in principle interfere with the measurements of the isobaric aldehydes pristanal and phytanal. Therefore, we chemically synthesized nonadecanal and eicosanal standards and determined their chromatographic characteristics. The two straight-chain aldehydes also resulted in two peaks each with the same mass transitions as pristanal and phytanal, but the straight-chain aldehydes eluted ∼1 min later. Next, extracts from *Arabidopsis* leaves (plants exposed to nitrogen deprivation) were analyzed by LC-MS/MS for the presence of C19:0 aldehyde-methyloximes (MRM 312/60) or C20:0 aldehyde-methyloximes (MRM 326/60). However, in both chromatograms, peaks were observed that eluted ∼0.5 min earlier than the standards, indicating that the long-chain aldehydes nonadecanal, eicosanal, pristanal, and phytanal could not be reliably identified in *Arabidopsis* leaves.

### Phytenal accumulates during chlorotic stress

**Figure 6:**
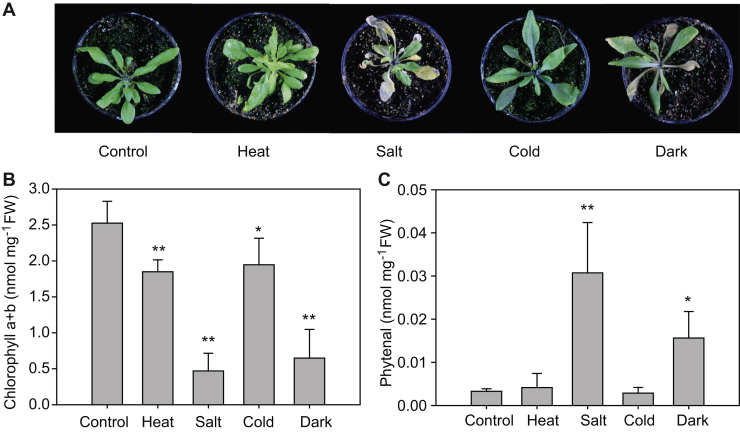
**Phytenal accumulation in *Arabidopsis* leaves under stress conditions.***A*, *Arabidopsis* WT plants were grown on MS medium for 2 weeks and then on soil for another 2 weeks. Subsequently, the plants were grown under control or different stress conditions for 10 days (heat, salt, cold, darkness, as outlined in Experimental procedures). *B*, chlorophyll was measured photometrically. Although the chlorophyll content remains high under heat and cold stress, it is degraded under salt and dark stress. *C*, phytenal was determined by GC-MS after methyloxime treatment. Phytenal accumulates to high amounts under salt and dark stress, that is, chlorotic stress conditions that lead to the hydrolysis of chlorophyll. Mean ± SD; n = 5; Student's *t* test; ∗*p* < 0.05; ∗∗*p* < 0.01.

To test whether phytenal accumulates under abiotic stress conditions, *Arabidopsis* WT plants were grown on soil and then exposed to dark, salt, heat, or cold stress. Chlorophyll was measured photometrically to determine whether the stress was accompanied with chlorophyll breakdown (chlorosis), and phytenal was quantified by GC-MS (Fig. 6). Salt or dark stress caused a decrease in the chlorophyll content, accompanied with an increase in phytenal. In contrast, heat or cold stress did not lead to large changes in chlorophyll contents, and at the same time, the amounts of phytenal remained low. These results indicate that phytenal accumulates only during chlorotic stress, likely by oxidation of phytol which is released by chlorophyll breakdown, but it does not increase under stress conditions that are not accompanied with chlorophyll breakdown.

### Phytol is converted into phytenal independently of light

**Figure 7:**
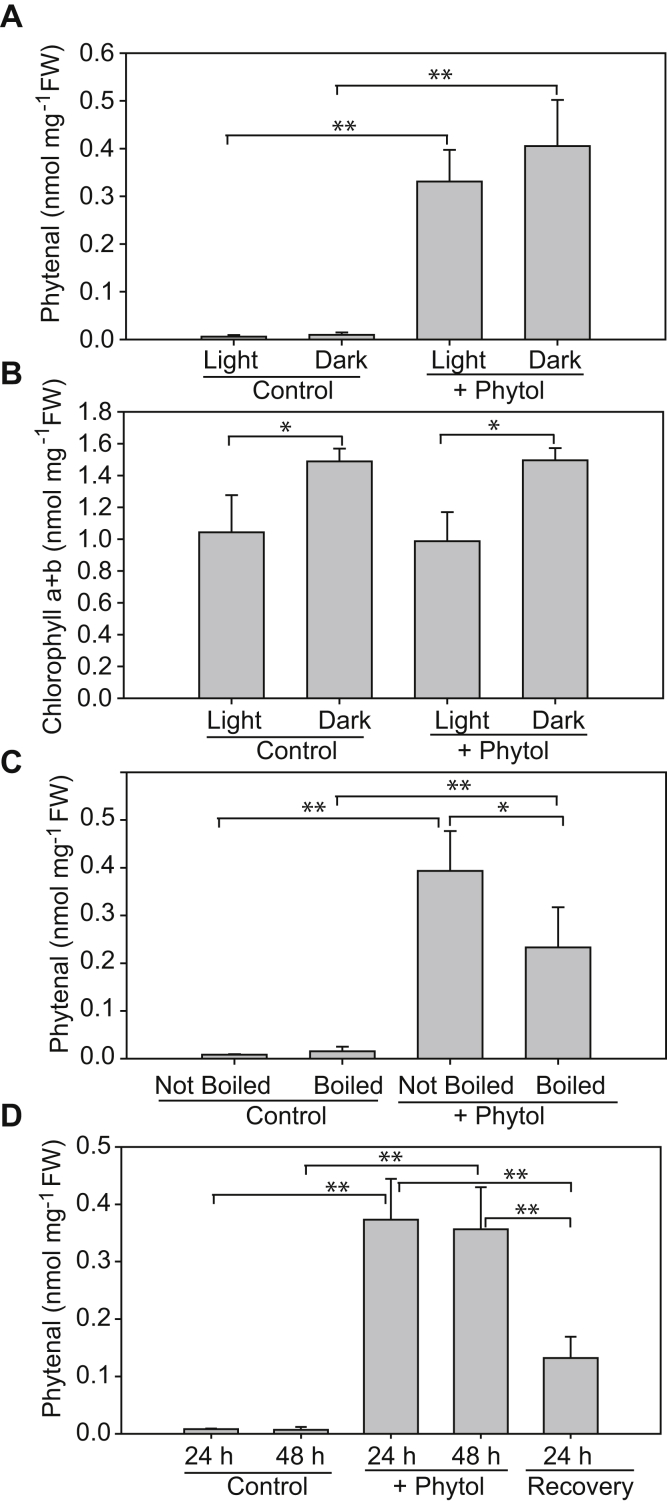
**Phytenal increases in *Arabidopsis* plants after phytol feeding.** WT Col-0 plants were grown on MS medium for 2 weeks before they were placed in the MES buffer (control), or MES buffer containing phytol (+phytol). *A*, the flasks were incubated in the light or dark for 24 h (16-h light/8-h dark) as indicated. Phytenal was measured by GC-MS after derivatization. *B*, chlorophyll was measured photometrically in the plants incubated in the light or dark. *C*, plants were directly placed into the flasks (not boiled) or heat-inactivated by boiling in the buffer at 95 °C for 5 min. The flasks were incubated for 24 h (16-h light/8-h dark). *D*, plants were incubated in control flasks or in the presence of phytol with 16-h light/8-h dark, for 24 or 48 h. For the recovery experiment, the plants incubated with phytol for 24 h were washed and incubated without phytol for an additional time of 24 h. Mean ± SD; n = 5; Student's *t* test; ∗*p* < 0.05; ∗∗*p* < 0.01.

To test the hypothesis whether free phytol can be converted into phytenal, whole *Arabidopsis* plants were incubated in the buffer (16 h light/8 h dark) in the presence of phytol. It has previously been shown that phytol can be taken up by *Arabidopsis* plants and that it can be used for the synthesis of FAPEs (6). During phytol feeding, the amount of phytenal increased to very high amounts without changing the chlorophyll content (Fig. 7). The increase in phytenal during phytol feeding was also observed when plants were incubated in the dark, again without a strong impact on the amount of chlorophyll. This finding indicates that phytol is oxidized to phytenal in the plant in a way independent of light, in contrast to previous findings (14).

Next, the plants were first killed by heat inactivation for 5 min at 95 °C to destroy all enzymatic activities. Incubation of the boiled plants in the presence of phytol still resulted in an increase in phytenal, albeit to a lesser extent than living, non–heat-inactivated plants. Therefore, the conversion of exogenous phytol to phytenal in the plants is in part mediated by nonenzymatic, chemical oxidation but might also be due in part to enzyme-dependent oxidation.

In addition, we studied the further metabolism of phytenal in a recovery experiment. Plants were incubated in the presence of phytol for 24 h. After washing, the plants were further incubated in the absence of phytol, and phytenal measured after 24 h. Figure 7*D* shows that the amount of phytenal was decreased to less than 50% after 24 h, indicating that it was metabolized, presumably by enzymatic oxidation (*e.g.*, aldehyde dehydrogenases).

### Phytenal is derived from phytol released during chlorophyll hydrolysis

**Figure 8:**
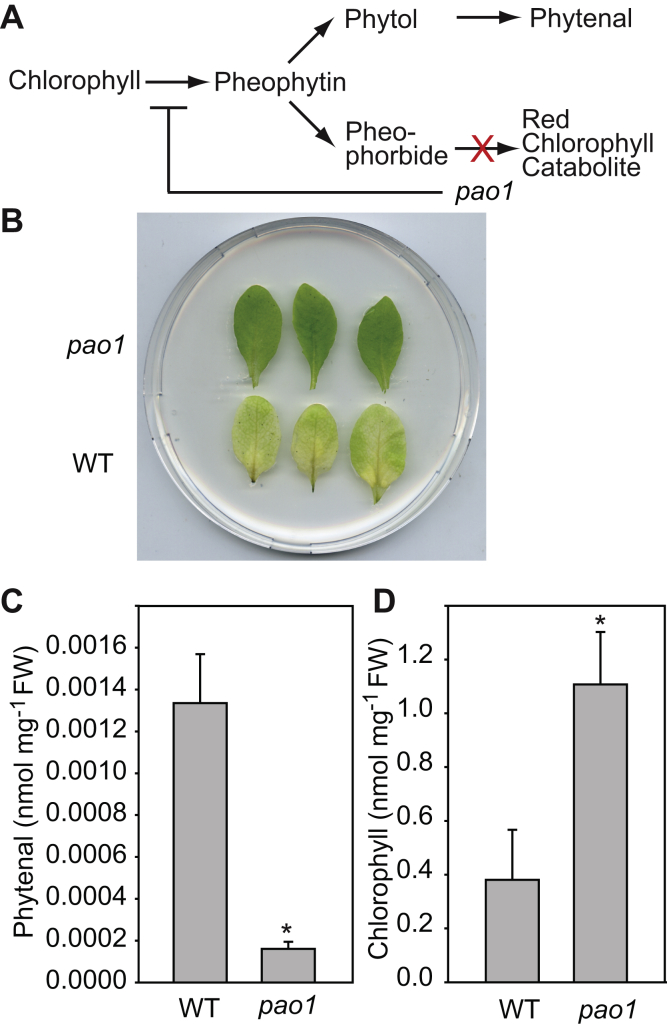
**Phytenal originates from phytol derived from chlorophyll degradation.***A*, PAO1 (pheophorbide a oxygenase) converts pheophorbide a into the red chlorophyll catabolite. In the *pao1* mutant, hydrolysis of chlorophyll a is downregulated. *B*, detached leaves of WT Col-0 and *pao1* mutant plants grown on MS medium for 2 weeks followed by growth on soil for 2 weeks were exposed to darkness for 7 days to induce senescence. The *pao1* leaves reveal a stay-green phenotype. *C*, phytenal and (*D*) chlorophyll were measured in the detached leaves after 7 days of dark exposure by GC-MS. The block in chlorophyll degradation in *pao1* is accompanied with reduced phytenal accumulation. Mean ± SD; n = 3; Student's *t* test; ∗*p* < 0.05.

The *pao1* mutant of *Arabidopsis* is deficient in pheophorbide a oxygenase, which converts pheophorbide a, the Mg-free form of chlorophyllide, into red chlorophyll catabolite (17). As a consequence, chlorophyll hydrolysis in the *pao1* mutant is suppressed, and the plants show a stay-green phenotype (Fig. 8) (8, 17). Detached leaves of WT Col-0 and *pao1* were dark-incubated for 1 week to stimulate senescence (Fig. 8). Subsequently, chlorophyll and phytenal were measured. WT leaves turned yellow and showed decreased chlorophyll amounts accompanied with increased phytenal levels. Leaves of the *pao1* mutant, however, remained green, and the chlorophyll content was not decreased. Furthermore, phytenal in *pao1* mutant leaves incubated in the dark did not increase. These results indicate that phytenal is derived from phytol originating from chlorophyll degradation.

### Role of phytenal in phytol metabolism

**Figure 9:**
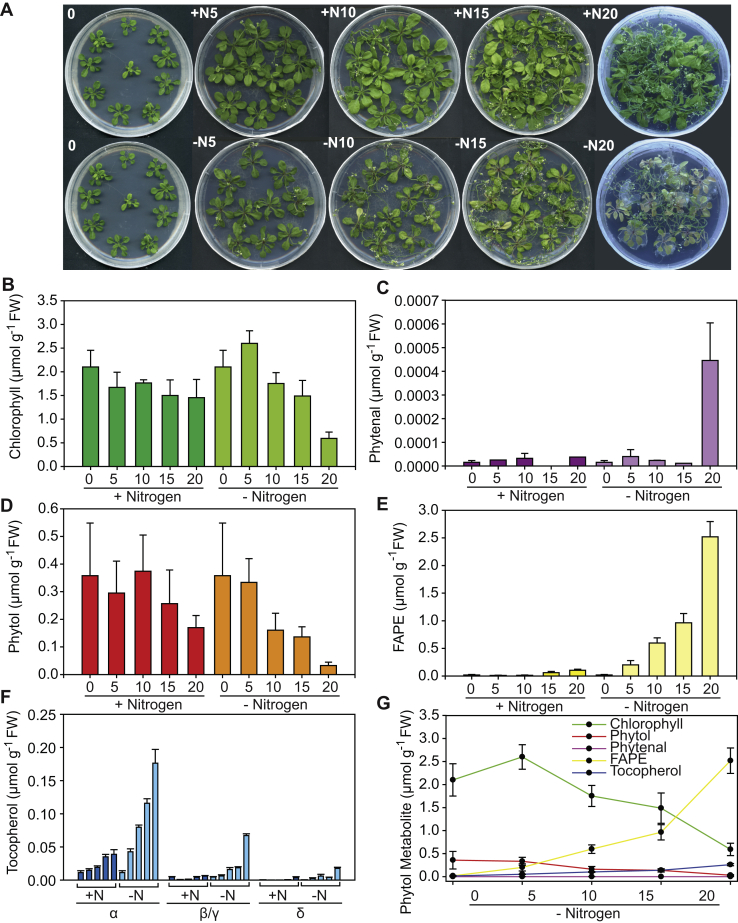
**Changes of phytol-derived metabolites during nitrogen deprivation.***A*, WT Col-0 plants were grown on MS medium for 2 weeks before they were placed on a nitrogen-containing medium (+N) or nitrogen-depleted medium (−N) for up to 20 days. The photos at day 0 show the same plants which are the control plants before starting the nitrogen-deprivation experiment. *B*, chlorophyll was measured photometrically. *C*, phytenal was quantified after derivatization with methyloxime by GC-MS. *D*, phytol was determined by GC-MS after trimethylsilylation. *E*, fatty acid phytyl esters (FAPEs) were determined by direct infusion Q-TOF MS. *F*, the different forms of tocopherol were quantified by reverse-phase HPLC. Note that β- and γ-tocopherol coelute and therefore were measured together. The individual *bars* show tocopherol amounts after 0, 5, 10, 15, and 20 on +N or −N plates. *G*, changes in the different phytyl-derived metabolites during nitrogen deprivation for up to 20 days. Q-TOF, quadrupole-TOF.

To study the role of phytenal during phytol metabolism, *Arabidopsis* WT Col-0 plants were first grown on Murashige and Skoog (MS) agarose medium and then transferred to a nitrogen-deprived medium. It is known that nitrogen deprivation stimulates chlorophyll breakdown accompanied with the release of phytol and an increase in tocopherol and FAPEs (6, 8). Lipids were isolated from the leaves for the measurements of chlorophyll, phytol, FAPEs, tocopherol, and phytenal at different time points. The amounts of chlorophyll and phytol decreased over the 20-day period of nitrogen deprivation (Fig. 9). On the other hand, the amounts of FAPEs, tocopherol, and phytenal increased during nitrogen deprivation (Figs. 2 and 4). The increase in phytenal levels was the strongest after 20 days of nitrogen deficiency. However, the levels of phytenal were still very low compared with the amounts of the other metabolites, indicating that phytenal constitutes a minor proportion of the total pool of phytol-derived compounds. Thus, while a substantial amount of phytol derived from chlorophyll degradation is deposited in the form of FAPEs and tocopherol, the steady-state levels of phytenal remain low. The total pool of phytol-derived metabolites remains almost unchanged over the time period of nitrogen deprivation (Fig. 9*G*). Therefore, most of the phytol which is released from chlorophyll during nitrogen deprivation is stored in the form of FAPE or converted into tocopherol, rather than being oxidized to phytenal.

### Phytol phosphorylation and phytol esterification compete with phytol oxidation

**Figure 10:**
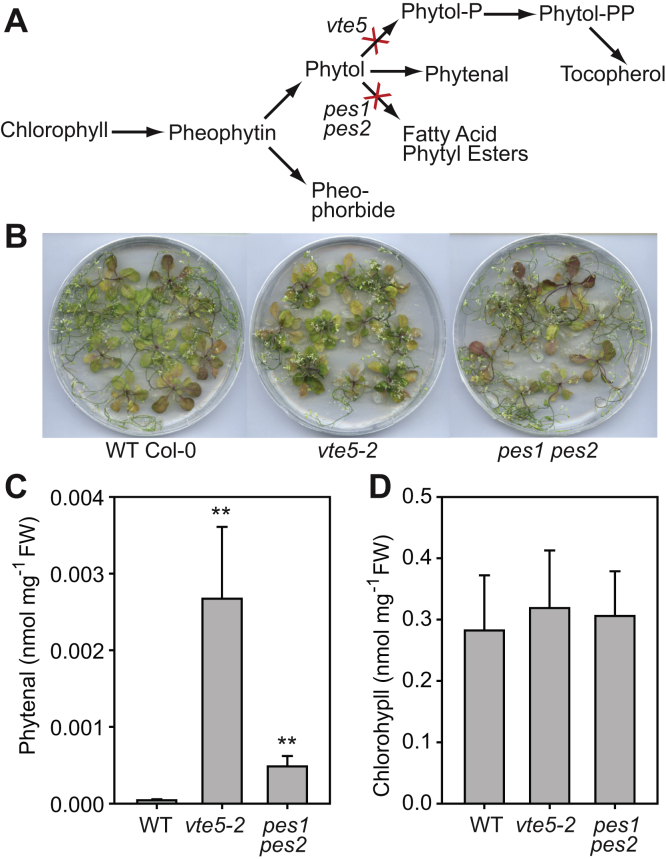
**Phytenal accumulation in the *vte5-2* and *pes1 pes2* mutants of *Arabidopsis* after nitrogen deprivation.***A*, the *vte5-2* mutant carries a block in phytol kinase involved in the production of phytol-P and phytyl-PP for tocopherol synthesis. The double-mutant *pes1 pes2* lacks phytyl ester synthesis activity. *B*, WT Col-0, *vte5-2*, and *pes1 pes2* mutant plants were grown on MS medium for 2 weeks before they were transferred to a nitrogen-deprived medium for an additional time of 2 weeks. *C*, phytenal and (*D*) chlorophyll were measured in plants after nitrogen deprivation by GC-MS or photometrically, respectively. Mean ± SD; n = 3; Student's *t* test; ∗∗*p* < 0.01.

FAPE synthesis is catalyzed by PES1 and PES2. Phytol phosphorylation which is mediated by VTE5 and VTE6, results in the synthesis of phytyl-PP which is the substrate for tocopherol synthesis (6, 7, 8). The two pathways depend on phytol that is released from chlorophyll. To test if phytenal synthesis competes with phytol esterification or phytol phosphorylation, phytol-derived metabolites were measured in the *pes1 pes2* and in the *vte5* mutant plants after nitrogen deprivation (Fig. 10). The *pes1 pes2* double mutant is almost completely devoid of FAPE because of a block in the two FAPE synthases PES1 and PES2 (6). The *vte5* mutant shows an ∼85% decrease in the tocopherol content in the leaves because of mutation in the phytol kinase VTE5 (7). After 20 days of nitrogen deprivation, the amounts of chlorophyll in *vte5* plants were similar to those in the WT, whereas chlorophyll was slightly decreased in *pes1 pes2*. Phytenal levels were strongly increased in the two mutant lines as compared with the WT, indicating that phytenal synthesis competes with the esterification and phosphorylation pathways for phytol.

## Discussion

In the present study, we established highly sensitive methods for the measurement of long-chain aldehydes in *Arabidopsis* leaves. One major long-chain aldehyde, that is, phytenal, was found, whereas other aldehydes were not reliably detected. Aldehydes are highly reactive because they form Schiff bases with amino group–containing compounds including amino acids and proteins. Therefore, plant cells presumably maintain the content of free aldehydes at a low concentration, possibly by action of aldehyde dehydrogenases, to avoid toxic interactions with other cellular components (18). Phytenal represents the oxidation product of phytol, and it was shown to be part of the phytol degradation pathway in humans (11, 15) (Fig. 1).

We considered the possibility that other long-chain aldehydes such as phytanal or pristanal potentially involved in phytol degradation could also be present in leaf extracts. Therefore, we used LC-MS/MS measurements with a Q-TOF or a triple quadrupole instrument to detect additional long-chain aldehydes in leaf extracts. However, pristanal and phytanal were barely detectable in the leaf. It is likely that phytanal is not an intermediate of phytol degradation, similar to the human pathway. Furthermore, free phytanic acid is not an intermediate of human phytol degradation, and phytanoyl-CoA, but not free phytanic acid, was detected in *Arabidopsis* plants, suggesting that the hypothetical branch pathway including phytanal and free phytanic acid is not used in plants (Fig. 1) (11, 12). On the other hand, pristanal might be rapidly degraded such that it cannot accumulate to detectable amounts.

Phytenal detected in *Arabidopsis* leaves is derived from endogenous phytol which in turn originates from chlorophyll breakdown. Therefore, phytenal represents a physiologically relevant intermediate of phytol degradation. This conclusion is based on the results that (i) phytenal accumulates during abiotic stresses which are accompanied with chlorophyll breakdown (salt, dark, nitrogen deprivation), but not during stresses that are not chlorotic (heat, cold) (Figs. 6, 9 and 10), (ii) the *pao1* mutant which shows a stay-green phenotype accumulates very low amounts of phytenal under stress (Fig. 8), and (iii) phytenal amounts increases in mutants where alternative routes of phytol metabolites are blocked (*vte5*, *pes1 pes2*) (Fig. 10). In this context, it should be noted that considerable amounts of phytenal accumulated not only after the chlorotic stresses indicated above but also after senescence stimulated by long (*i.e.*, 7–10 days), dark incubation of whole plants (Fig. 6) or detached leaves (Fig. 8). On the other hand, a 24-h dark incubation of whole plants did not cause senescence but rather led to an increase in the chlorophyll content (Fig. 7). This finding is in agreement with previous results indicating that short dark incubation of whole plants suppresses senescence, whereas dark treatment of individual leaves stimulates senescence (19, 20)

The amount of phytol or phytenal detected during chlorophyll breakdown in *Arabidopsis* WT leaves remained rather low, compared with the total chlorophyll content (Fig. 9). After 20 days of nitrogen deprivation, >80% of chlorophyll was degraded, but the contents of phytol and phytenal remained very low. In addition, the amounts of tocopherol were low, while FAPEs strongly increased, indicating that under these conditions, most of the phytol derived from chlorophyll breakdown was deposited in the form of esters. Nitrogen deprivation possibly represents a transient stress where phytol is deposited in an intermediate pool of phytyl esters which is available for recycling when growth conditions improve (8). However, the finding that the steady-state levels of phytenal are low does not exclude the possibility that it represents an authentic intermediate of phytol catabolism.

The finding that the *pes1 pes2* or *vte5* mutants show increased phytenal contents under nitrogen deprivation indicates that free phytol not used for esterification or phosphorylation can be oxidized to phytenal. This finding shows that removal of phytol from metabolism *via* esterification or phosphorylation is important to keep the concentration of phytenal in the plant cell low.

Phytol externally added to the plants is taken up and can be used for FAPE synthesis (6). We observed a strong increase in phytenal in plants after phytol feeding, indicating that phytol is taken up and oxidized in the plants. The increase in phytenal was independent of light as it also occurred in complete darkness (Fig. 7). Therefore, the conversion of phytol into phytenal in plants is not mediated *via* photooxidation (14). Interestingly, the oxidation of phytol to phytenal was also observed with plants which were heat-inactivated before the feeding experiment. However, the conversion of phytol into phytenal was reduced approximately one-third after heat inactivation. Therefore, a large proportion of phytol is presumably oxidized in the plant tissue independently from enzymes. It cannot be excluded that phytol is oxidized by other components in the plant cell, for example, metabolites. Thus, the mechanism of the oxidation of phytol to phytenal and its further metabolism in the plant remain to be analyzed.

## Experimental procedures

### Arabidopsis lines and growth conditions

The *Arabidopsis* WT Col-0, *pao1* (deficient in chlorophyll degradation), *vte5-2* (deficient in tocopherol), and *pes1 pes2* mutants (deficient in FAPE synthesis) were described previously (6, 8, 17). *Arabidopsis* plants were grown for 14 days on Murashige and Skoog medium and then transferred to soil for growth of another 2 weeks (21). Plants were grown under a 16-h light/8-h dark regime at 22 °C and a light intensity of 150 μmol s^−1^ m^−2^.

For abiotic stress experiments, the plants growing on soil were incubated under dark, salt, heat, or cold stress. Dark stress was implemented by placing the plants into complete darkness in the growth chamber. For salt stress, plants were repeatedly (day 0, 3, 6, 9) watered with 200-mM NaCl. Heat stress was performed by placing the plants at 60 °C for 1 h each on 4 days (day 0, 3, 6, and 9). The plants were cold-stressed by transferring them to a growth chamber at 4 °C. Leaves were harvested 10 days after initiating the stress (dark, salt, heat, cold). For nitrogen deprivation, plants were germinated on MS medium with 1% sucrose. After 3 weeks, plants were transferred to a synthetic medium with or without nitrogen (5).

### Standards

Hexadecanal (palmitic aldehyde, 16:0al) was obtained from Cayman Chemical. Phytol (*cis*- and *trans*-isomers) was obtained from Sigma Aldrich. Phytenal was synthesized by oxidation of phytol with pyridinium chlorochromate (22, 23). Briefly, a solution of phytol and pyridinium chlorochromate (in a nanomolar ratio of 0.01–0.09) in dichloromethane was stirred for 90 min. The reaction mixture was passed through a silica column. The reaction product (phytenal) was collected in the flow-through and eluted with 5-ml dichloromethane. Pristanol was synthesized from pristanic acid (Larodan) as follows. Pristanic acid methyl ester was produced by methylation with 1 N methanolic HCl at 80 °C for 30 min. The methyl esters were extracted after addition of 1 ml 0.9% NaCl and 1-ml hexane. The hexane was evaporated and the methyl ester reduced to pristanol with LiAlH_4_ (24). Pristanal (pristyl aldehyde) was obtained from pristanol by oxidation with pyridinium chlorochromate as described for phytenal. Nonadecanal (19:0al), eicosanal (icosanal, 20:0al), and phytanal were produced from the corresponding fatty acids (nonadecanoic acid, eicosanoic acid, from Sigma Aldrich; phytanic acid from Larodan). The fatty acids were converted into their methyl esters and then reduced to the alcohols with LiAlH_4_, and the alcohols oxidized with pyridinium chlorochromate as described above.

### Supplementation of plants with phytol

Plants were grown on MS medium with 1% sucrose. After 3 weeks, plants were transferred to flasks containing MES-KOH buffer, pH 6.5. Phytol and Tween-20 were added to final concentrations of 1% and 0.2% (v/v), respectively. Flasks were incubated for 24 h in a 16-h light/8-h dark regime. For incubation in the dark, some flasks were wrapped with aluminum foil.

### Chlorophyll, tocopherol, phytol, and FAPE measurements

Chlorophyll was measured photometrically (25). Tocopherol was analyzed by HPLC using a reverse-phase column and a fluorescence detector (26). FAPEs were quantified by direct infusion MS (8). Free phytol was trimethylsilylated with N-methyl-N-(trimethylsilyl)trifluoroacetamide and measured by GC-MS using octadecenol (oleyl alcohol; Sigma Aldrich) as the internal standard (8).

### Extraction, derivatization, and purification of long-chain aldehydes

Leaves (100 mg) were frozen in liquid nitrogen and homogenized with stainless-steel beads in a Precellys homogenizer (Bertin Instruments). To the homogenate, 1-ml chloroform/methanol (2:1, v/v) and 10-nmol hexadecanal (internal standard for GC-MS) and 500 μl of 300-mM ammonium acetate were added (27, 28). Samples were vortexed, centrifuged for 3 min at 5000*g*, and the organic phase collected. Samples were extracted again with 1 ml of chloroform and the organic phases combined. The solvent was evaporated under a stream of nitrogen gas. The aldehydes were derivatized with 100-μl methylhydroxylamine hydrochloride (methoxylamine hydrochloride) (Alfa Aesar/Thermo Fisher) in pyridine (20 mg/ml) for 1 h at room temperature (15). The pyridine was evaporated, the samples were dissolved in 1 ml of hexane, and loaded onto an SPE column (100 mg of silica; Phenomenex). The column was washed with 3x 1 ml of hexane. Long-chain aldehyde-methyloximes were eluted with 3 ml of hexane/diethylether (99:1, v/v). The solvent was evaporated under a flow of nitrogen. The samples were dissolved in 100-μl hexane and transferred to GC autosampler vials.

### GC conditions

The aldehyde-methyloximes were analyzed with an Agilent GC-MS instrument using an Agilent HP-5MS column (30 m length) with helium as the carrier gas at a flow rate of 7 ml min^−1^. The oven was set at the following temperatures: initial, 70 °C; ramp with 5 °C min^−1^ to 310 °C; hold for 1 min at 310 °C; ramp to 70 °C with 5 °C min^−1^.

For the determination of the recovery, a defined amount of phytenal was mixed with a leaf lipid extract followed by derivatization, SPE, and quantification by GC-MS. The amount of phytenal measured after mixing with the leaf extract was compared with the phytenal content of a sample containing derivatized phytenal without the leaf lipid extract and without SPE and the ratio calculated. The reproducibility of the phytenal analytical method was determined by comparing the phytenal levels in two independent quantification experiments performed on two different days. The linearity of phytenal quantification was measured by quantifying different amounts of phytenal in the presence of the same amount of the lipid extract (100 mg of leaves) and internal standard (hexadecanal). The limit of detection was defined for peaks detectable with a signal-to-noise ratio of 3:1.

### Measurements of aldehydes by LC-MS

Aldehydes were extracted in the presence of 1 nmol hexadecanal (internal standard, converted into methyloxime derivatives and purified by SPE as described above). The solvents were evaporated with nitrogen gas and dissolved in acetonitrile. Aldehyde–methyloximes (10 μl) were injected on a reverse-phase column (Eurospher II C8, 150 × 3 mm, 3 μm, with precolumn; Knauer). The flow rate was 0.5 ml min^−1^. The initial eluent (from 0 to 2 min) was 20% buffer A (H_2_O/acetonitrile/formic acid, 63:37:0.02, v/v/v) and 80% buffer B (100% acetonitrile). The content of buffer B was raised to 100% until 12 min, held at 100% until 25 min, and then reduced to 80% at 32 min. Aldehyde–methyloximes were analyzed by electrospray ionization coupled with a quadrupole-TOF (Agilent 6530 Accurate Mass Q-TOF) mass spectrometer. The ions were measured in the positive mode with declustering potential of 70 V and collision energy of 45 V.

For quantification, the samples were measured by LC-MS/MS using the same column and solvent gradient coupled to a Q-Trap 6500+ mass spectrometer (Sciex) in positive-ion mode by MRM with a declustering potential of 70 V. The dwell time was 100 ms. The mass transitions for the different straight-chain and branched-chain aldehyde-methyloximes are given in Table S1.

## Data availability

All data described are contained within the article.

## Supporting information

This article contains supporting information.

## Conflict of interest

The authors declare that they have no conflicts of interest with the contents of this article.
