# Supplementary material for: Phytol derived from chlorophyll hydrolysis in plants is metabolized via phytenal
Source: J Biol Chem. 2021 Mar 11;296:100530. doi: 10.1016/j.jbc.2021.100530 (PMC8054155; doi:10.1016/j.jbc.2021.100530)
Supplement: Supplemental Table S1 [file mmc1.docx]

**Supporting Table 1. Parameters for detection of aldehyde-methyloximes by multiple reaction monitoring using LC-MS/MS measurements with the Q-Trap instrument.**

| Abbreviation | Aldehyde | Q1 Mass (m/z) | Q3 Mass (m/z) | Dwell Time  (ms) |
| --- | --- | --- | --- | --- |
| 10:0al |  | 186.19 | 60.04 | 100 |
| 11:0al |  | 200.20 | 60.04 | 100 |
| 12:0al |  | 214.22 | 60.04 | 100 |
| 13:0al |  | 228.23 | 60.04 | 100 |
| 14:0al |  | 242.25 | 60.04 | 100 |
| 15:0al |  | 256.26 | 60.04 | 100 |
| 15:3al | Farnesal | 250.22 | 60.04 | 100 |
| 16:0al | Hexadecanal | 270.28 | 60.04 | 100 |
| 17:0al |  | 284.30 | 60.04 | 100 |
| 18:0al |  | 298.31 | 60.04 | 100 |
| 19:0al | Nonadecanal/  Pristanal | 312.33 | 60.04 | 100 |
| 20:0al | Eicosanal/  Phytanal | 326.34 | 60.04 | 100 |
| 20:1al | Phytenal | 324.33 | 96.08 | 100 |
| 20:4al | Geranylgeranal | 318.28 | 60.04 | 100 |
